# Development of an anoikis-related gene signature and prognostic model for predicting the tumor microenvironment and response to immunotherapy in colorectal cancer

**DOI:** 10.3389/fimmu.2024.1378305

**Published:** 2024-05-08

**Authors:** Chuanchang Li, Junyong Weng, Le Yang, Hangjun Gong, Zhaolong Liu

**Affiliations:** ^1^ Department of General Surgery, Shuguang Hospital, Shanghai University of Traditional Chinese Medicine, Shanghai, China; ^2^ Department of Colorectal Surgery, Fudan University Shanghai Cancer Center, Shanghai, China; ^3^ Department of Gastrointestinal Surgery, Shuguang Hospital, Shanghai University of Traditional Chinese Medicine, Shanghai, China

**Keywords:** Anoikis, colorectal cancer, tumor microenvironment, immunotherapy, metastasis

## Abstract

The effect of anoikis-related genes (ARGs) on clinicopathological characteristics and tumor microenvironment remains unclear. We comprehensively analyzed anoikis-associated gene signatures of 1057 colorectal cancer (CRC) samples based on 18 ARGs. Anoikis-related molecular subtypes and gene features were identified through consensus clustering analysis. The biological functions and immune cell infiltration were assessed using the GSVA and ssGSEA algorithms. Prognostic risk score was constructed using multivariate Cox regression analysis. The immunological features of high-risk and low-risk groups were compared. Finally, DAPK2-overexpressing plasmid was transfected to measure its effect on tumor proliferation and metastasis *in vitro* and *in vivo*. We identified 18 prognostic ARGs. Three different subtypes of anoikis were identified and demonstrated to be linked to distinct biological processes and prognosis. Then, a risk score model was constructed and identified as an independent prognostic factor. Compared to the high-risk group, patients in the low-risk group exhibited longer survival, higher enrichment of checkpoint function, increased expression of CTLA4 and PD-L1, higher IPS scores, and a higher proportion of MSI-H. The results of RT-PCR indicated that the expression of DAPK2 mRNA was significantly downregulated in CRC tissues compared to normal tissues. Increased DAPK2 expression significantly suppressed cell proliferation, promoted apoptosis, and inhibited migration and invasion. The nude mice xenograft tumor model confirmed that high expression of DAPK2 inhibited tumor growth. Collectively, we discovered an innovative anoikis-related gene signature associated with prognosis and TME. Besides, our study indicated that DAPK2 can serve as a promising therapeutic target for inhibiting the growth and metastasis of CRC.

## Introduction

CRC ranks as the second leading cause of cancer-related mortality globally. The incidence of CRC continues to rise, with an estimated 1.9 million new cases in 2020 (1). Although primary tumors can be radically resected at the early-stage, approximately 20% of patients with CRC present with metastasis at initial diagnosis, and up to 50% will develop metastases after surgery (2, 3). Novel therapeutic approaches, such as chemotherapy and immunotherapy have exhibited promising results in enhancing the survivability of CRC patients. However, their efficacy for individuals with distant metastases remains restricted. This underscore the need to develop innovative and precise biomarkers for early diagnosis of CRC.

Anoikis is a specialized type of apoptosis triggered by the loss of cell adhesion to the extracellular matrix (4). Following detachment from ECM or neighboring cells, cancer cells migrate to distant locations, where they then re-attach and proliferate (5). Previous studies have documented that tumor cells can evade anoikis to facilitate invasion and metastasis. These processes may be achieved through activating lipid rafts, modulating the acidic conditions in the tumor microenvironment (TME), or inducing reactive oxygen species (ROS) production (6–8). Inhibiting the resistance of tumor cells to anoikis can effectively hinder tumorigenesis and metastasis. Eckhardt BL et al. discovered that enhanced BMP4 expression sensitizes cancer cells to anoikis, reducing the abundance of circulating tumor cells and restraining breast cancer metastasis (9). Through analyzing the characteristics of anoikis-related genes, studies have predicted the prognosis of clear cell renal cell carcinoma (10), hepatocellular carcinoma (11), and osteosarcoma (12). Although targeting anoikis holds promise as an novel approach in cancer therapy, further studies are needed to clarify the role of ARGs in CRC.

The TME serves as a key regulator in tumor progression. It encompasses various immune cells such as helper T cells, regulatory T cells, dendritic cells, tumor-associated macrophages, cancer-associated fibroblasts (CAFs), and associated pathways that induce inflammation (13–15). Recent studies have highlighted the clinical and pathological significance of TME in predicting prognosis and therapeutic efficacy. Several innovative strategies, including immune checkpoint blockade, metabolic inhibitors, and gut microbial metabolism, were shown to regulate TME (16–18). Immune cells in the tumor microenvironment, such as macrophages and T cells regulate tumor progression, metastasis, and susceptibility to immunotherapy (19, 20). Hu JL et al. found that CAFs contribute to metastasis and drug resistance by secreting miR-92a-3p (15). It is imperative to thoroughly investigate the infiltration patterns of the TME to develop an optimal therapeutic strategy.

Death-associated protein kinase 2 (DAPK2) belongs to the family of death-associated protein kinases and acts as a calcium/calmodulin-dependent kinase (21). DAPK2 participates in apoptosis, autophagy, myeloid differentiation, and erythropoiesis (22, 23). Extensive researches have been conducted concerning the association of DRAK2 with the development of type 1 diabetes and non-alcoholic fatty liver disease (24, 25). Moreover, DAPK2 is involved in the development of various types of cancer, including acute myeloid leukemia (26), cutaneous T cell lymphoma (27), and breast cancer (28). Recent studies have found that DRAK2 also activates T-cells in the lymphoid system (29). Nevertheless, research on the role of DAPK2 in tumors is still limited.

In this research, we systematically profiled the expression of 1057 CRC samples to assess the molecular patterns of ARGs. Further analyses confirmed the close relationship between the three anoikis clusters and the infiltration characteristics of TME and prognosis. Three gene clusters were identified based on the anoikis-related differentially expressed genes (DEGs). Afterwards, a risk score system was developed to serve as a biomarker for evaluating prognosis and guiding targeted therapy and immunotherapy in patients with CRC. Ultimately, the role of the anoikis-regulatjng gene DAPK2 in suppressing tumor growth and metastasis was confirmed both *in vitro* and vivo.

## Materials and methods

### Data collection for CRC

The original transcriptome and clinical data of 530 TCGA-colonic adenocarcinoma (COAD)/rectum adenocarcinoma (READ) samples were obtained from the TCGA database (https://portal.gdc.cancer.gov/). Moreover, GSE39582 (GPL570 platform, 585 CRC tissue samples) was downloaded from the GEO database (https://www.ncbi.nlm.nih.gov/geo/). Then, fragments per million kilobase (FPKM) values of the TCGA sample were transformed into transcripts per million kilobase (TPM) values. The package ‘sva’ was used to merge the TCGA and GEO data, normalize the data, and eliminate batch effects.

### Identifying prognostic anoikis regulators and analyzing their characteristics

A total of 32 anoikis regulators were acquired from the MSigDB database (http://www.gsea-msigdb.org/gsea/index.jsp) (30). First, we analyzed the relationship between anoikis regulators and prognosis in CRC patients using the R package ‘Survminer’, and obtained 18 prognostic ARGs. Then the genetic loci, somatic mutation rate, and CNV of prognostic ARGs were analyzed.

### Consensus clustering analysis for anoikis regulators

Unsupervised consensus cluster analysis was used to analyze the molecular characteristics. Using the R package ‘ConsensusClusterPlus’ individuals were divided into three groups. Then, the study compared subgroups to investigate differences in clinicopathological features including gender, age, and T, N, and M stages.

### Functional enrichment and immune infiltration

DEGs between subtypes were screened using the R package ‘limma’ with the cut-off criteria of | logFC| ≥ 1 and *p*< 0.05. To calculate the biological functions of different anoikis clusters, gene set variation analysis (GSVA) was performed. The gene ontology (GO) enrichment analysis was performed using the ‘clusterProfiler’ package. To quantify the abundance of immune cell infiltration, ssGSEA was performed. *P<* 0.05 was considered statistically significant. To investigate DAPK2-related molecular characteristics and pathways, GSEA analysis was performed by the ‘ClusterProfiler’ R package using GSEA on-line analysis tool (http://www.bioinformatics.com.cn). ‘Canonical pathways’ was used as a reference gene set. P< 0.05 and FDR< 0.25 was considered statistically significant.

### Establishment of anoikis gene signature

Univariate Cox regression analysis was used to filtering the DEGs, and 489 prognostic DEGs were obtained. Candidates were optimized using LASSO-penalized Cox regression to prevent overfitting. Finally, multivariate Cox regression analysis was conducted to screen the potential genes that were used to establish the anoikis risk score. The risk score was computed as follows:


$$Risk\;Score=\sum_{i=1}^{n}Coefi*expri$$

The variable ‘n’ represents the number of prognostic DEGs in the risk model. ‘expr’ represents the corresponding expression, and ‘Coef’ represents the regression coefficient (31).

### Nomogram establishment and validation

The nomogram model was formulated using the ‘RMS’ package, integrating both the risk score of anoikis and clinicopathological characteristics. The calibration and receiver operating characteristic (ROC) curves were generated to evaluate the prediction accuracy and dependability of the nomogram model.

### Somatic mutation and immunological features analysis

Immune checkpoint genes were examined in the different anoikis risk score groups. The microsatellite instability (MSI) in the high-risk and low-risk groups was analyzed. Additionally, we assessed the anti-CTLA4/PD-1 medication IPS scores in high-risk and low-risk group. The IPS data was retrieved from the Cancer Immunome Atlas (https://tcia.at/).

### Drug susceptibility analysis

In order to evaluate the predictive capability of risk scores in drug sensitivity, we utilized the ‘pRRophetic’ package to compute and contrast the IC50 values (50% inhibitory concentration) of various anticancer drugs among cohorts with high-risk and low-risk scores.

### Cell culture

Nine human CRC cell lines (HCT15, RKO, DLD1, HCT116, NUM460, HT29, SW1116, HCT8, SW480, and SW620) were obtained from the Cell Bank of Shanghai Institute of Biological Sciences, Chinese Academy of Sciences (Shanghai, China). Cells were cultured in RPMI 1640 medium (Gibco, China, USA) with 10% fetal bovine serum (Gibco, USA) in a humidified incubator with 5% CO_2_ at 37°C. The medium was refreshed every two days.

### Clinical samples

This investigation harvested 67 pairs of CRC specimens and adjacent normal tissues from Shuguang Hospital Affiliated to Shanghai University of Traditional Chinese Medicine. All participants afforded written informed consent. The study was approved by the Ethics Committees of the hospital. Detailed information about the included patients and their clinical features can be found in **Supplementary Table S6**.

### RT-PCR and Western blotting

TRIzol reagent (Invitrogen, USA) was used to extract total RNA from CRC tissues and cells. Quantitative RT-PCR was performed using the TB Green Fast qPCR Mix (Takara, RR430B, Japan) on the ABI 7500 system (Applied Biosystems, American). We utilized the 2^-ddCt^ method to calculate the fold change of DAPK2, and the results were normalized using GAPDH levels. The primers used in this study are presented in **Supplementary Table S7**.

Cell lysis was achieved using a combination of lysis buffer (Beyotime, China) and protease inhibitor (Roche, USA), facilitating the extraction of proteins. Western blotting analysis was conducted using the following antibodies: Anti-DAPK2 (Abcam, ad151601), anti-GAPDH (Abcam ab128915), anti-Caspase-3 (Abcam ab184787), anti-Caspase-7 (Abcam ab32522), anti-Cleaved-Caspase-3 (Abcam ab214430), anti-Cleaved-Caspase-7 (Abcam ab256469), Anti-AKT1 (Abcam ab235958), Anti-p-AKT1 (Abcam ab133458), Anti-CyclinD1 (proteintech 26939-1-AP), Anti-Snail antibody (Cell Signaling Technology #4719), Anti-E-cadherin (Abcam ab227639), and Anti-N-cadherin (Abcam ab18203). To begin, 15% sodium dodecyl sulphate-polyacrylamide gel (SDS-PAGE) electrophoresis was performed to transfer the protein bands onto the polyvinylidene fluoride (PVDF) membrane. The membrane was blocked with 5% fat-free milk and incubated with related diluted primary antibody (1:2000) at 4°C for 6 hours. Subsequently, the protein was incubated with diluted secondary antibody (1:10,000) at room temperature for 2 hours. Finally, the ECL Western blotting substrate (Thermo Scientific) was used to facilitate the development of the membrane.

### Plasmid construction and transfection

To enhance the expression of DAPK2, the lentiviral vector CMV (OBIO scientific service, Shanghai, China) was used to insert full-length DAPK2 or control sequences at the EcoRI and XbaI loci. Subsequently, lentivirus containing the CMV-DAPK2 plasmid (DAPK2-OE group) and the CMV-MCS plasmid (Vector group) were transfected into SW1116 cells.

### Cell proliferation assay

According to the manufacturer’s instructions, cell viability was measured using the Cell Counting Kit-8 (Beyotime Biotechnology, China). 2000 cells were seeded in 96-well plates and the CCK8 reagent was introduced to each well at the same time every day for five days. Absorbance at 450nm is measured after incubation for a specified time. To perform the colony formation assay, we plated 500 cells in each well of 6-well plates. Once distinct colonies were formed, we washed the cells with PBS and stained them with 0.1% solution of crystal violet for 5 minutes. Subsequently, the number of visible colonies was counted. Cell apoptosis was detected using the Annexin V-FITC/PI apoptosis detection kit (Beyotime Biotechnology, China) in accordance with the manufacturer’s instructions. Flow Jo software was used to analyze the collected data.

### Transwell assay and wound healing assay

After 24 hours of plasmid transfection, 5 × 10^3^ cells were inoculated into each chamber of the Transwell plate and cultured overnight. We randomly selected 5 fields of view for invasive cell counting under the light microscope after staining. The wound healing assay was performed according to the manufacturer’s instructions.

### Animal experiments

Male thymus BALB/c nude mice were purchased from the Shanghai Zoological Center of the Chinese Academy of Sciences and raised in specific pathogen-free environments at animal laboratory center of Shanghai University of Traditional Chinese Medicine. The 5-week-old naked mice were classified into two groups at random (n = 3 per group). 0.1 ml 0f cell suspension containing 2 × 10^6^ cells was injected into each mouse. Volume = length × width ^2 × 0.5 was the formula used to measure the size of tumors. All animal experiments were carried out in compliance with relevant regulations and guidelines of the Experimental Animal Ethics Committee, Shanghai University of Traditional Chinese Medicine.

### Statistical analysis

All bioinformatics data were analyzed using R 4.1.2 version. Data were reported as mean ± standard deviation. T-test was used for comparisons between two groups, while the ANOVA method was used to compare more than two groups. The *X^2^* test was utilized for comparing count data. Statistical analysis for these experiments was conducted using GraphPad Prism 7.0 (GraphPad Software, USA) and SPSS 20.0 (SPSS Inc., USA) software. Statistical significance was defined as *p<* 0.05.

## Results

### Identification of prognostic values, expression, genetic variation and function of 18 anoikis regulators

**Figure 1:**
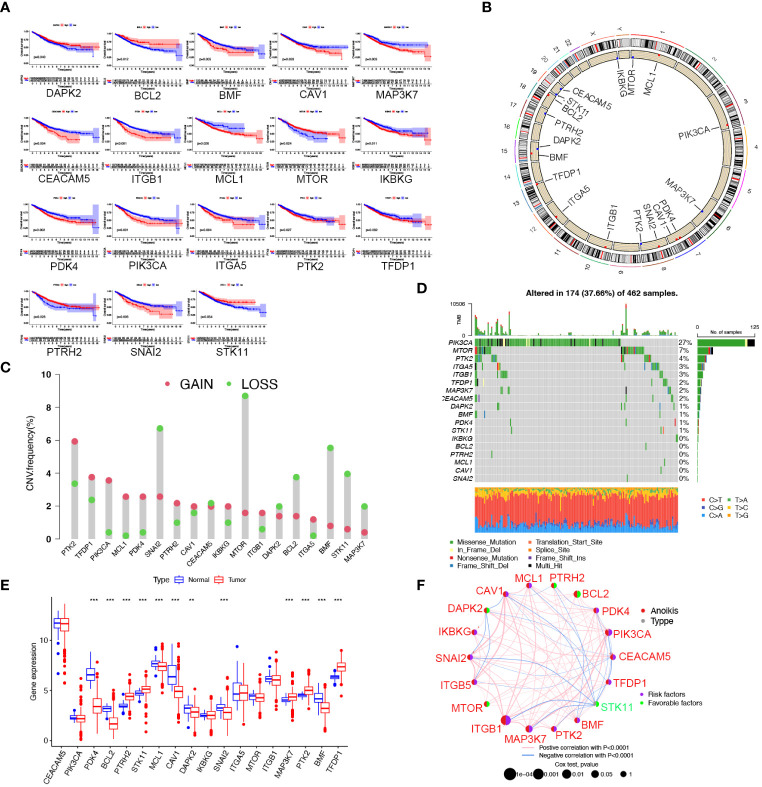
Landscape of the 18 ARGs in CRC: prognostic values, expression, and genetic variants. **(A)** The overall survival of 18 ARGs in CRC. **(B)** The positions of ARGs’ CNV changes. **(C)** Frequency of CNV. **(D)** The frequency of ARG mutations. **(E)** The different expression of the 18 ARGs between tumor tissue and normal tissue from the TCGA database (** *p* < 0.01; ***p< 0.001). **(F)** The interactions between ARGs in CRC. Positive correlations are denoted by red strings and negative correlations are denoted by blue strings. The intensity of the correlation is indicated by the shades of color.

In this research, we obtained 32 anoikis regulators from the MSigDB database. After integrating clinical data and expression profiles from TCGA-COAD, TCGA-READ, and GSE39582 samples, we analyzed the relationship between 32 ARGs and OS (**Supplementary Table S1**). The expression levels of 18 ARGs were significantly associated with the overall survival of patients (**Supplementary Table S2**). Specifically, patients with high expression of DAPK2, BCL2, STK11, MTOR, and PTRH2 exhibited prolonged survival times. (**Figure 1A**). The position of copy number variation (CNV) alteration of prognostic ARGs on the chromosomes in TCGA-CRC database is shown in **Figure 1B**. Furthermore, investigating CNV variations uncovered a widespread occurrence of CNV mutations in 18 regulators of anoikis. Amplification of copy numbers was observed in PTK2, TFDP1, PIK3CA, PDK4, and MCL1, while SNAI2, MTOR, BCL2, BMF, MAP3K7, and STK11 exhibited significant deletions (**Figure 1C**). We extensively examined the prevalence of somatic mutations in the 18 ARGs in CRC. Notably, PIK3CA displayed the highest mutation frequency, reaching up to 27%, whereas the mutation rates for other genes were considerably lower (**Figure 1D**). Besides, we discovered notable variations in the expression of 12 ARGs between tumor and normal samples based on TCGA data. PTRH2, STK11, MAP3K7, PTK2, and TFDP1 were upregulated in CRC, while PDK4, BCL2, MCL1, CAV1, DAPK2, SNAI2, and TMF were downregulated (**Figure 1E**). A network plot extensively visualizes the intricate associations, predictive significance, interactions, and connections among ARGs in CRC (**Figure 1F**).

To investigate the biological functions of 18 ARGs, we conducted a KEGG enrichment analysis using the Metascape database (https://metascape.org/). The results displayed that the anoikis regulators were involved in the negative regulation of anoikis, the signaling pathway of PI3K/Akt, and the promotion of cell migration (**Supplementary Figure S1**).

### Correlation of anoikis pattern with TME infiltration, functional enrichment, and clinical traits

**Figure 2:**
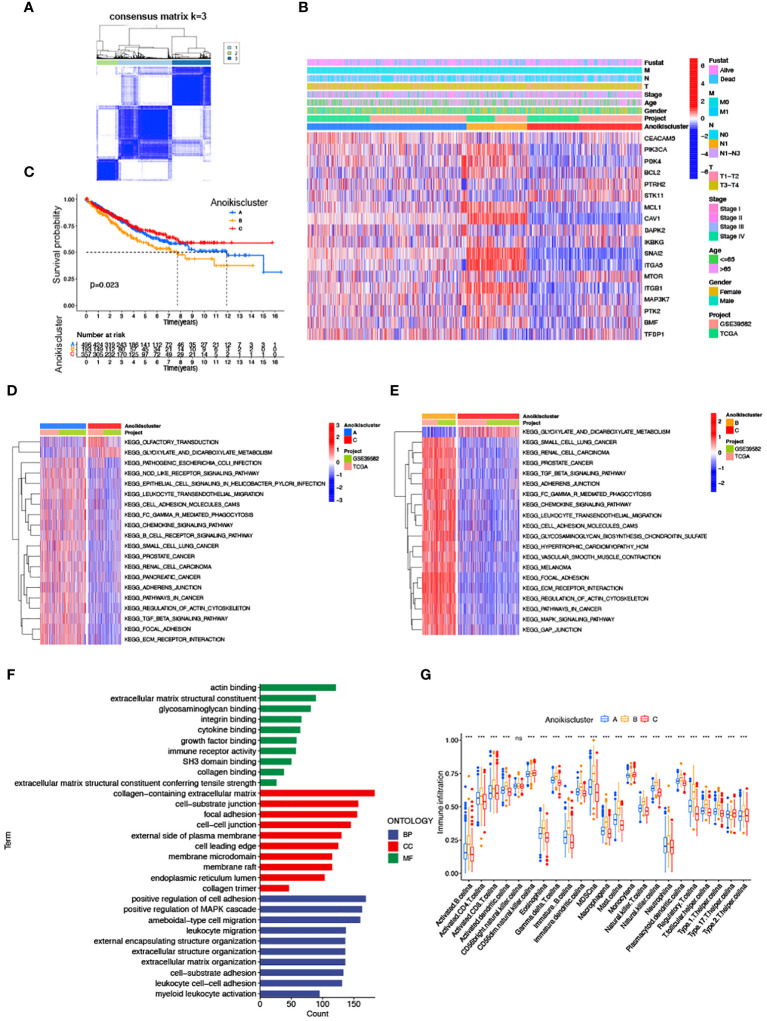
Correlation of anoikis pattern with TME infiltration, functional enrichment, and clinical traits. **(A)** Molecular subgroup screening by means of unsupervised consensus cluster analysis. **(B)** Expression level of ARGs and clinicopathological features in different subtypes. **(C)** Subtype-specific K-M OS curves. **(D, E)** GSVA of two Anoikisclusters related cellular pathways, with red and blue representing activated and inhibited pathways. **(D)** A *vs* C, **(E)** B *vs* **(C, F)** GO analyses of prognostic DEGs. **(G)** Correlations between immune cell infiltration levels in three Anoikisclusters (*** *p*< 0.001).

We subdivided 1057 CRC samples into three clusters (K=3), termed Anoikiscluster A, B and C (**Figure 2A**). The heatmap visualized the specific expression of ARGs in the A, B and C clusters (**Figure 2B**). Furthermore, Anoikiscluster C exhibited a strong association with lower stages and fewer deceased states. The Kaplan-Meier curve showed a significant discrepancy between the overall survival of the three clusters. Notably, patients in the Anoikiscluster C exhibited a longer overall survival compared to those in Anoikiscluster A and B (**Figure 2A**, *p* = 0.023). GSVA enrichment analysis was performed to unravel the biological functions of the three groups. Tumor pathways and tumors, such as small cell lung cancer, prostate cancer, and renal cell carcinoma, were found to be less enriched in Anoikiscluster C compared to Anoikiscluster B and A. Conversely, the Anoikiscluster B and A showed a significant increase in the TGF-beta signaling pathway, adherens junction, ECM receptor interaction, focal adhesion, cell adhesion molecules (CAMs), and actin cytoskeleton regulation (**Figures 2D, E**). Interestingly, the glyoxylate and dicarboxylate metabolism was remarkably abundant in subtype C. The DEGs were screened among the three Anoikisclusters using the ‘limma’ R algorithm and 3038 DEGs were identified (**Supplementary Figure S2**, **Supplementary Table S3**). The GO enrichment analysis was conducted to unravel the biological behaviors of these DEGs. The findings indicated that these genes are primarily involved in cell adhesion. This includes various aspects, such as extracellular matrix containing collagen, junction between cells and substrate, junction between cells, facilitation of cell adhesion, and binding to actin (**Figure 2F**). This aligns with the previous findings. We used the ssGSEA analysis to assess associations between the three subtypes and 23 immune cells, and unveil the role of ARGs in the TME. According to our findings, there was a significant difference in the level of immune cell infiltration among the 3 clusters (**Figure 2G**). Anoikiscluster C was predominately enriched in Th17 cells and monocytes compared with Anoikiscluster A and B.

### Identification of gene subtypes based on anoikis related prognostic DEGs

**Figure 3:**
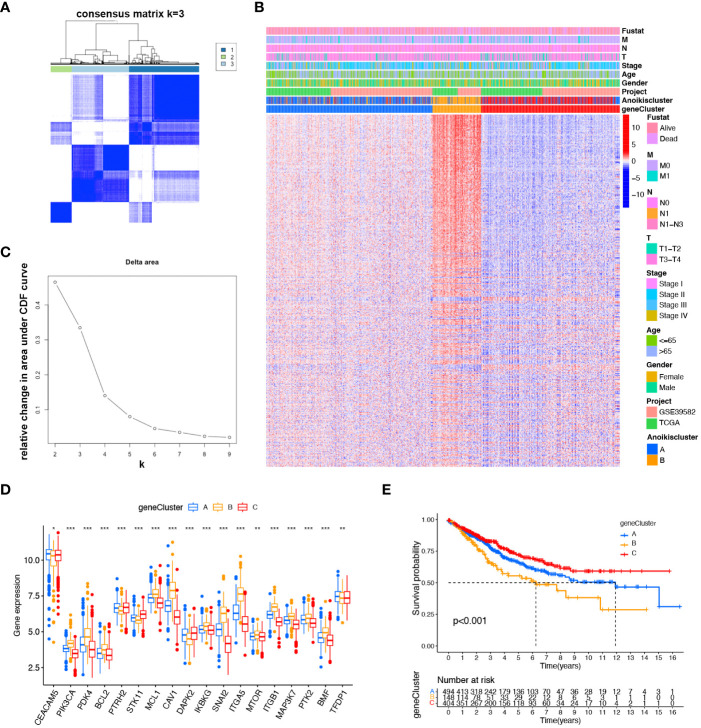
Identified gene subtypes based on DEGs. **(A)** Consensus matrix heatmap identified three clusters (k = 3). **(B)** Relative change area under cumulative distribution function curve. **(C)** Differences in clinicopathologic characteristics of the three geneClusters. **(D)** Variations in the expression of the 18 ARGs in three geneClusters. * *p* < 0.05 ** *p*< 0.01, *** *p*< 0.001. **(E)** K-M OS curves of three geneClusters.

To more comprehensively analyze the genetic characteristics of DEGs, we employed the unsupervised consensus cluster analysis based on the expression of 489 prognostic DEGs (**Supplementary Table S4**). The results indicated that CRC patients can be classified into three subcategories using - k = 3: A (n = 494), B (n = 148), and C (n = 404) (**Figures 3A, B**). Moreover, we investigated the relationship between clinical features and genetic subtypes. GeneCluster C was associated with a higher survival rate, a greater number of individuals with Anoikiscluster C, and a lower stage when compared to geneCluster A and B (**Figure 3C**). Confirming our expectations in regard to the anoikis pattern, the expression of ARGs displayed significant variation among the three geneClusters (**Figure 3D**). The K-M curve demonstrated that patients with geneCluster C exhibited the longest overall survival, whereas those with geneCluster B experienced the shortest overall survival (*p<* 0.001) (**Figure 3E**).

### Construction of the prognostic signature based on DEGs

**Figure 4:**
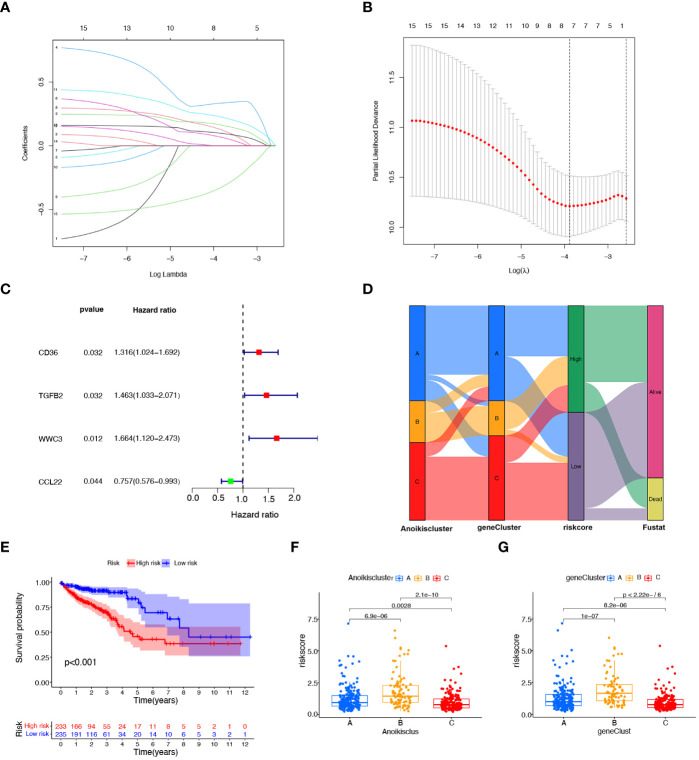
Construction of the prognostic signature based on prognostic DEGs. **(A)** The LASSO coefficient spectrum of prognostic DEGs. **(B)** Partial likelihood bias identified by the LASSO regression model. **(C)** The forest map of prognostic DEGs constructed by multivariate Cox regression analysis. **(D)** Alluvial diagram of subtype distributions among groups, risk scores, and survival states. **(E)**, K−M curves showing the differences in survival between the high-risk and low- risk groups. **(F)** Variations in risk scores among different Anoikisclusters. **(G)** Variations in risk scores among different geneClusters.

To explore the prognostic value of DEGs in CRC, 27 prognostic DEGs were screened out by univariate Cox regression analysis in the TAGA dataset. To select a robust and effective risk model for prognosis prediction, we performed the LASSO regression analysis and multivariate Cox regression analysis on these DEGs. The optimal gene combination was screened by LASSO regression analysis (**Figure 4A**) and partial likelihood deviance (**Figure 4B**). Subsequently, we conducted multivariate Cox regression analysis to constructed a prognostic signature consisting of 4 genes: CD36, TGFβ2, WWC3 and CCL22 (**Figure 4C**; **Supplementary Table S5**). The risk score of each patient was calculated as follows (**Supplementary Table S5**): risk score = CD36 × (0.250124947) + TGFβ2 × (0.329147201) + WWC3 × (0.400373998) + CCL22 × (-0.499379016). All CRC patients were categorized into high-risk and low-risk subtypes equally based on the median risk score. Patients were split into two cohorts by the risk model, a training group (n = 236) and a testing group (n = 232). Independent analyses were performed on both the training and testing sets to analyze the survival rates and the distribution of the risk scores. To differentiate high-risk and low-risk groups, the risk scores were computed and a median threshold of 1 was established (**Supplementary Figure S3A**). The two cohorts’ survival scatter diagrams demonstrated that mortality rates rose with risk scores (**Supplementary Figure S3B**). Survival analysis revealed that patients with a low-risk score had a better prognosis than those with a high-risk score (**Supplementary Figure S3C**).

**Figure 4D** illustrates the proportion of patients among the two risk score groups, the three Anoikisclusters, and the three geneClusters. The K-M survival curve showed that the high-risk score group had a substantially worse prognosis than the low-risk group (**Figure 4E**). Subsequently, the association between the risk score and the Anoikiscluster and the gene subtypes was confirmed using the Kruskal-Wallis test. Statistically significant differences were observed in risk score among distinct Anoikisclusters. Anoikiscluster C exhibited a considerably reduced risk score when compared to Anoikiscluster A and B (**Figure 4F**). Moreover, geneCluster B presented the maximum risk score, whereas geneCluster C displayed the minimum risk score (**Figure 4G**). These findings are consistent with the outcomes depicted in **Figures 2C**, **3E**.

### Developing a nomogram with independent prognostic value

**Figure 5:**
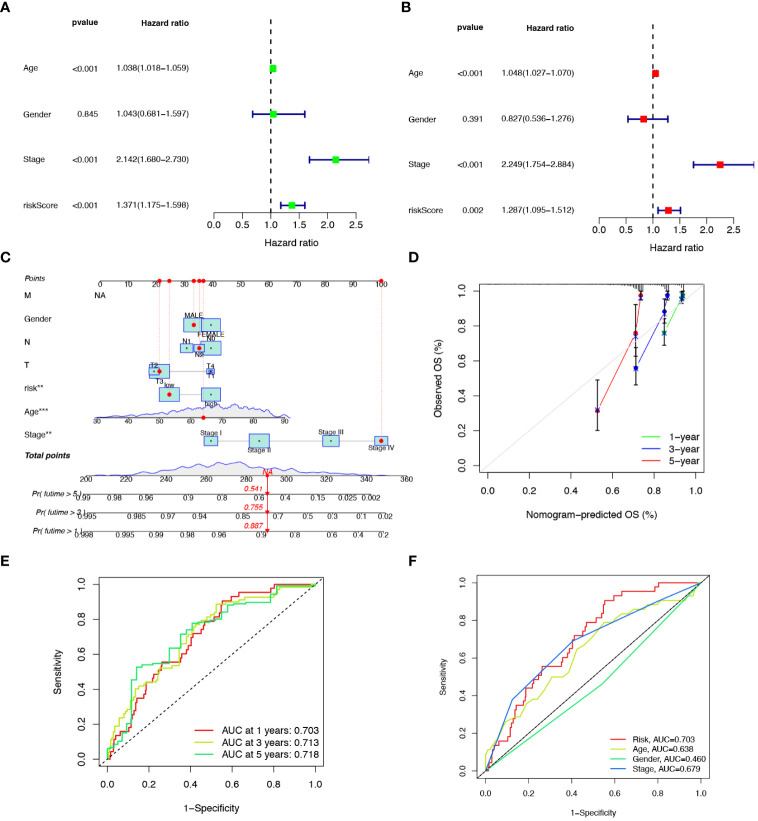
Developing a nomogram with independent prognostic value. Univariate **(A)** and multivariate **(B)** Cox regression analysis of the prognostic value of anoikis-related risk score and clinicopathological parameters. **(C)** Construction of the nomogram based on the risk score to evaluate OS of 1-, 3-, and 5-years for CRC patients. **(D)** Calibration curve for the nomogram. **(E)** Prediction of AUC for forecasting 1-, 3-, and 5-year OS. **(F)** ROC curves for the risk score and clinical characteristics.

To explore the prognostic factors in CRC, we conducted univariate and multivariate Cox regression analyses, incorporating variables, such as risk score, age, sex, and pathological stage. The results of both univariate (**Figure 5A**) and multivariate (**Figure 5B**) analyses indicated that the risk score isa significant and independent prognostic factor for CRC patients. To establish a quantitative method for predicting the prognosis of CRC patients, we developed a nomogram by incorporating these prognostic aspects (**Figure 5C**). The calibration curves provided sufficient overlap between the observed 1-, 3-, and 5-year survival probabilities, affirming the precision of our nomogram (**Figure 5D**). Furthermore, the predictive power of the nomogram was demonstrated through ROC analysis, showcasing notable prediction accuracy. Specifically, the ROC values for the 1-year, 3-year, and 5-year periods were 0.703, 0.713, and 0.718, respectively (**Figure 5E**). To evaluate the accuracy of survival prediction by the risk score, we calculated the Area Under the Curve (AUC). The results indicated that the AUC of risk score (AUC = 0.736) was higher than that of other clinicopathological characteristics (**Figure 5F**).

### Comparison of response to immunotherapy between subgroups

**Figure 6:**
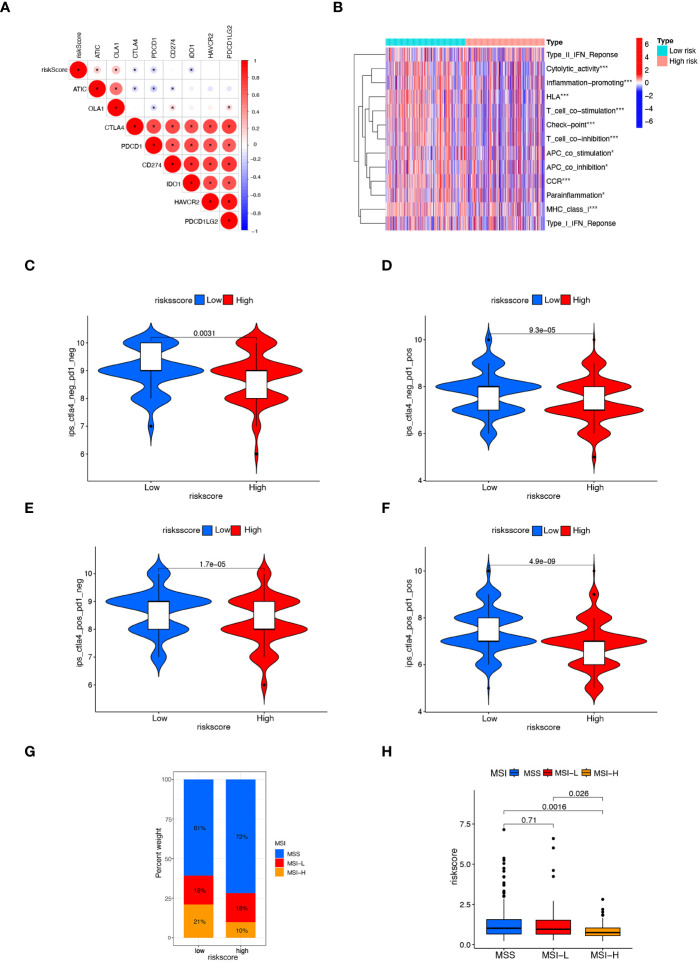
Comparison of response to immunotherapy between subgroups. **(A)** Correlation between risk score and the expression of immune checkpoint genes in CRC patients. **(B)** Heatmap displaying immunological function enrichment for high-risk and low-risk score groups. * *p*< 0.05, and *** *p*< 0.001. **(C–F)** Comparison of CTLA4/PD-1 IPS scores between high-risk group and low-risk group. CTLA4–PD1–, CTLA4– PD1+, CTLA4+ PD1–, and CTLA4+ PD1+. **(G, H)** The relationship between MSI and risk scores, *** *P*< 0.001.

MSI is a biological markers of tumor response to immunotherapy, and immunotherapy is beneficial for patients with higher MSI-H status. The tumor immune landscape was analyzed based on the anoikis related risk score in CRC patients. Initially, we examined the expression of several immune checkpoint genes. It was observed that in the low-risk score group, the expression of immune checkpoint genes such as CTLA4, PDCD1, and IDO1 was significantly higher compared to the high-risk group. Conversely, the high-risk group manifested notably lower expression levels of immune checkpoint genes like ATIC and OLA1 (**Figure 6A**). Although the PD-L1 expression was higher in the low-risk group than in the high-risk group, the difference was not statistically significant (**Supplementary Figure S4**). Immune-related functions analysis showed that check point, T cell co-inhibition, T cell co-stimulation, inflammation promoting, and cytolytic activity were markedly upregulated in the low-risk group (**Figure 6B**).

The IPS score, a novel biomarker for tumor response to immunotherapy, is a valuable tool for assessing the efficacy of anti-PD1 and anti-CTLA4 therapies. It has been observed that patients with higher IPS scores tend to exhibit higher immunogenicity, resulting in better response to immunotherapy (32). Patients with a low risk score exhibited a notably higher IPS score (**Figures 6C–F**), suggesting that patients with low-risk CRC may respond better to immunotherapy with PD-1 and CTLA4. Lower risk scores were found in the MSI-H group compared to the MSS and MSI-L groups (**Figures 6G, H**). These findings suggest the higher efficacy of immunotherapy for CRC patients with the low-risk scores.

### Evaluation of anticancer drugs sensitivity

The differences in sensitivity to anticancer drugs between the low- and high-risk score subtypes were evaluated to determine if the risk score can guide treatment decision-making. According to the IC50 values, that CRC patients with low risk score exhibited a higher sensitivity to the common chemotherapy drugs, such as Oxaliplatin, Bortezomib, and Temozolomide (**Supplementary Figures S5A–C**). Additionally, individuals with low risk scores showed a greater response to targeted medications, such as dabrafenib (BRAF inhibitor), AZD8055, and ribociclib (**Supplementary Figures S5D–F**). These findings suggest that the risk score may provide invaluable information for treating CRC.

### Detecting the expression of anoikis gene DAPK2

**Figure 7:**
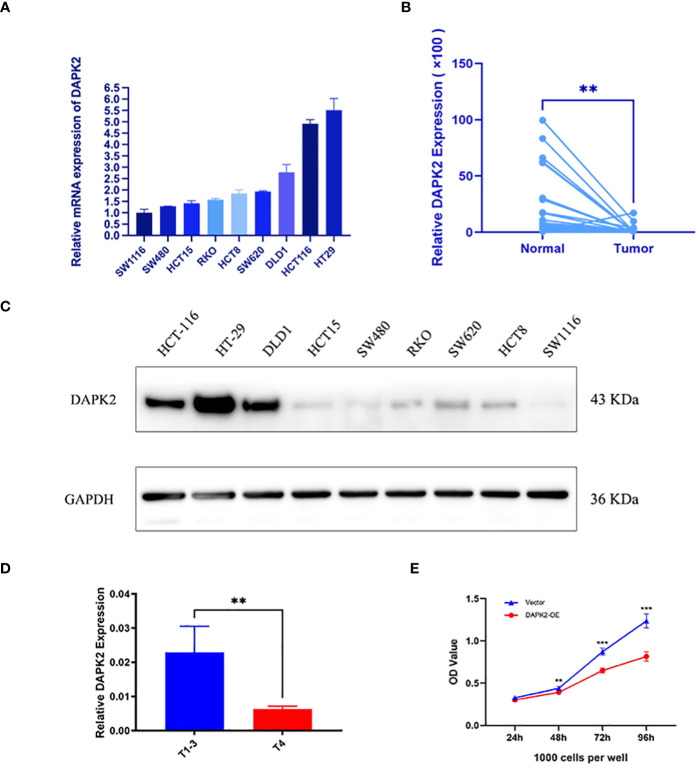
The expression of the anoikis-related gene DAPK2. **(A)** RT-PCR detected the mRNA expression of DAPK2 in 9 CRC cell lines. **(B)** RT-PCR detected DAPK2 mRNA expression in cancer and paracancerous tissues. **(C)** Western blotting analysis of the DAPK2 protein expression in 9 CRC cell lines. **(D)** The bar chart showed the relationship between DAPK2 and T stage. **(E)** CCK8 assessing the reproductive capacity in the vector group and DAPK2 overexpression group of SW1116 cells. ** *p* < 0.01 and *** *p* < 0.001.

Our previous analysis indicated that high expression of the DAPK2 can serves as a molecular marker of good prognosis. To investigate the anti-tumor effect of DAPK2, we first detected the expression of DAPK2 in 9 CRC cell lines includingHCT15, RKO, DLD1, HCT116, NUM460, HT29, SW1116, HCT8, SW480, and SW620. The results of RT-PCR and Western blotting showed that HT29 cells exhibited the highest expression of DAPK2, while SW1116 cells had the lowest expression of DAPK2 (**Figures 7A, C**). Subsequently, we assessed the expression of DAPK2 in both malignant and adjacent non-malignant tissues from 67 individuals with CRC. DAPK2 was substantially downregulated in the tumor samples compared to the paracancerous group (**Figure 7B**). The expression of DAPK2 was negatively correlated with T stage of the tumor. Compared to T4 patients, T1-3 patients exhibited a significantly elevated expression of DAPK2 (**Figure 7D**).

Overexpression of DAPK2 promoted cell apoptosis, and inhibits cell proliferation and migration.

**Figure 8:**
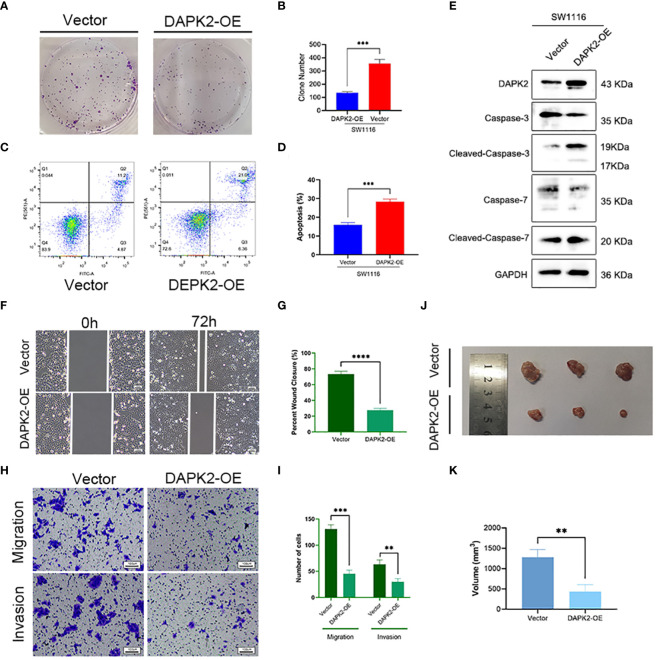
Overexpression of DAPK2 promoted cell apoptosis and inhibited cell proliferation. **(A)**. Representative pictures of colony formation. **(B)** The average number of cell colonies in **(A)** (*** *p*< 0.001). **(C)** Flow cytometry showing the percentage of cells in each quadrant. Cells in quadrants Q2 and Q3 represented apoptotic cells. **(D)** Cell apoptotic rate of **(C)** (*** *p*< 0.001). **(E)** Western blotting showing the protein levels of DAPK2, Caspases-3, Cleaved-Caspase-3, Caspases-7 and Cleaved-Caspase-7. **(F)** Representative images of SW1116 cells in wound healing assay. Scale bar, 100 um. **(G)** The wound closure rate in **(F)**, *** *p*<0.01. **(H)** Representative images of SW1116 cells in Transwell plates. Scale bar, 100 um. **(I)** Quantification of the number of migration and invasion cells in. **(J)** Images of xenograft tumors displayed the shape and size of tumor. **(K)** Volume of xenograft tumors in **(J)** at day 32 (n = 3, ** *p*<0.01).

We conducted additional experiments to confirm the tumor suppressor role of DAPK2 both *in vitro* and *in vivo*. To overexpress DAPK2, we transfected DAPK2 stable transfection plasmids into SW1116 cells. The control group received transfection of an empty plasmid. Subsequently, we executed a CCK8 assay and observed that the proliferation ability of cells was significantly decreased after DAPK2 overexpression (**Figure 7E**). The results of the colony formation assay visually indicated that the DAPK2 overexpression group exhibited a reduced number of cell clones compared to the control group (**Figure 8A**). Statistical analysis also confirmed a significant disparity in the number of clones between the two groups (**Figure 8B**). We then compared the apoptosis rate between the two groups. In the vector group, 11.2% of cells were in Q2 and 4.87% of cells were in Q3. In contrast, the DAPK2 overexpression group had 21.0% of cells in Q2 and 6.36% of cells in Q3. The apoptosis rate of the DAPK2 overexpression group was significantly higher compared to the control group (**Figures 8C, D**) (*p*< 0.001). Western blotting revealed that DAPK2 expression significantly increased after transfection with the overexpression plasmid. Additionally, the expression of cleaved-caspase-3 and cleaved-caspase-7 were significantly higher in the overexpression group than in the control group, while the expressions of caspase-3 and capase-7 were significantly lower in the overexpression group(**Figure 8E**). Subsequently, wound healing assay and Transwell assay proved that high DAPK2 expression restrained the migration and invasion of SW1116 (**Figures 8F–I**). The cell-derived xenograft model demonstrated that the tumor volume in DAPK2 overexpression group was significantly smaller compared to the control group (**Figure 8J**). This difference was statistically significant (*p<* 0.01) (**Figure 8K**).

### Overexpression of DAPK2 inhibited the migration of CRC cells by blocking AKT1/CyclinD1 pathway

**Figure 9:**
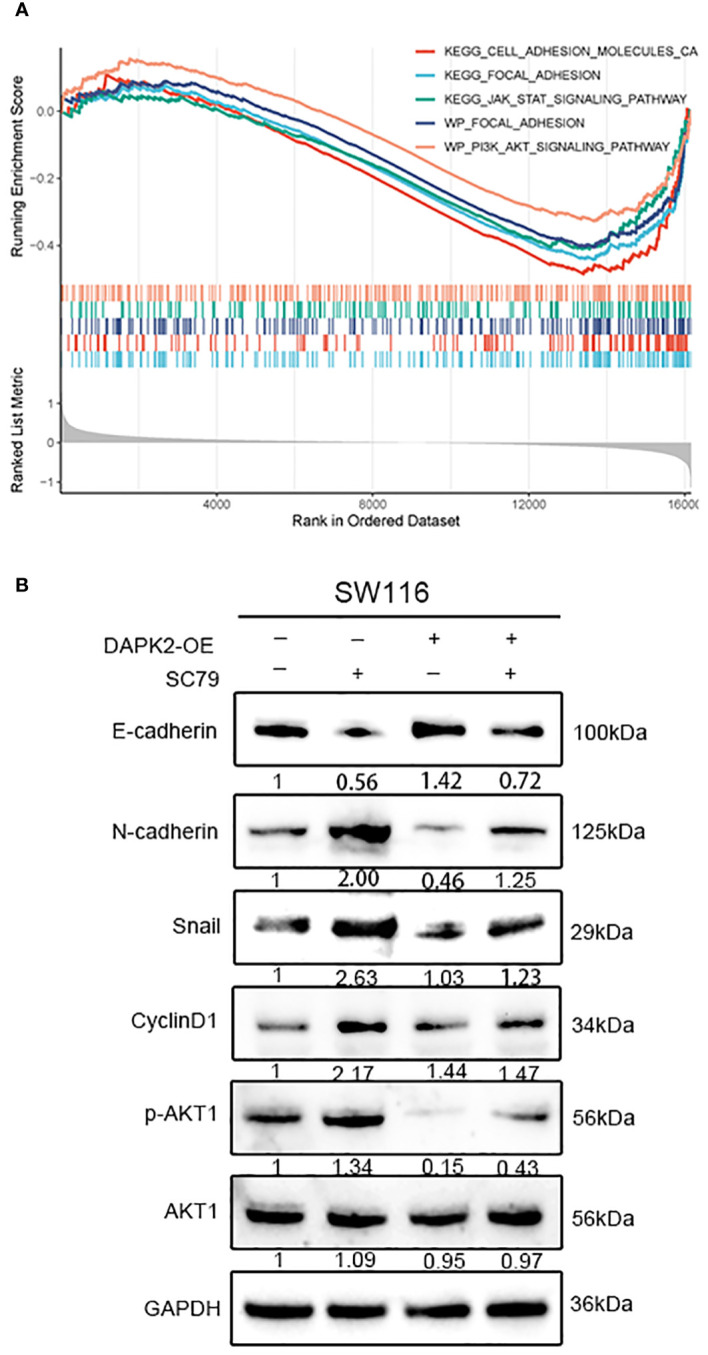
Overexpression of DAPK2 inhibited AKT1/CyclinD1 pathway. **(A)** GSEA plots for 5 DAPK2 related pathways significantly enriched in TCGA and GEO database. Screening criteria of select pathways: FDR-value< 0.25, and p-value< 0.05. **(B)** SW1116 cells were treated with DAPK2 overexpression plasmid or AKT1 agonist (SC79). The expression of AKT1, p-AKT1 and epithelial and mesenchymal markers were assessed via western blotting.

To investigate the potential mechanism of DAPK2 suppresses the metastasis of CRC, we categorized patients with CRC from the TCGA and GEO database based on DAPK2 expression levels. The GSEA analysis was conducted to identify pathways affected by DAPK2, revealing significant enrichment in CELL/ADHESION/MOLECULES, JAK/STAT/SIGNALING/PATHWAY, PI3K/AKT/SIGNALING/PATHWAY, and FOCAL/ADHESION pathways (**Figure 9A**; **Supplementary Table S8**). After careful analysis of genes associated with the PI3K/AKT pathway, we hypothesized that DAPK2 may act through the AKT1/CyclinD1 pathway. Western blotting confirmed that increased DAPK2 expression decreased AKT1 phosphorylation without affecting total AKT1 levels. Additionally, the DAPK2-overexpression group exhibited higher E-cadherin levels and lower levels of CyclinD1, Snail, and N-cadherin compared to the control group. However, AKT1 agonist (SC79) significantly promoted AKT1 phosphorylation and weekend the effect caused by DAPK2 (**Figure 9B**). These results suggest that DAPK2 overexpression suppresses CRC cell migration through the AKT1/CyclinD1 pathway.

## Discussion

Anoikis occurs when cells lack proper connections to the ECM. Anoikis plays a key role in normal development and tissue homeostasis, while anoikis resistance is critical for cancer invasion and metastasis (30). Inhibition of anoikis resistance has an important role in tumor treatment (33, 34). However, the associations between anoikis regulators and molecular patterns, clinicopathological subtypes, prognostic value, TME infiltration characteristics, and response to immunotherapy remained unknown. Therefore, clarifying the role of anoikis-related molecular patterns can advance our understanding of anoikis and its characteristics in CRC, and help explore the potential of ARGs as markers for predicting prognosis and anti-tumor immune response.

In this study, we identified 18 prognostic anoikis regulators. They are involved in the pathway that regulate anoikis and cell migration. We identified three different anoikis subtypes based on the expression of these genes. Anoikiscluster C was associated with a lower stage and fewer states of death, which is consistent with the longer overall survival of patients in the Anoikiscluster C than those in the Anoikiscluster A and B. We then obtained the intersection genes of the three clusters and performed GO analysis on these genes. These intersecting genes were mainly involved in cell adhesion and migration. During the epithelial-mesenchymal transition, the structural adhesion between epithelial cells becomes weak, enhancing mobility and aggressiveness of cancer cells. Regulation of cell polarity, organization of the cytoskeleton, expression of vein filaments and down-regulation of cytokeratins are involved in such phenomenon (35). The ssGSEA analysis indicated a significant infiltration of immunosuppressive cells and innate immune cells, including macrophages, regulatory T cells, Th1 cells, and Th2 cells in the Anoikiscluster B. Previous studies have found that the induction of immune tolerance by Th2 cells undermines the efficacy of cancer immunotherapy (36). Macrophages can polarize into distinct phenotypes based on the characteristics of TME. Specifically, they can differentiate into M1 macrophages, which possess pro-inflammatory and anti-tumor properties, or M2 macrophages, which possess anti-inflammatory and immunosuppressive activities (37, 38). M2 polarization of macrophage in the TME can promote CRC development and metastasis (39, 40). Despite the high abundance of immune cells in Anoikiscluster B, its prognosis remains unfavorable. Previous studies have indicated that these cells assume an immunosuppressive function. They encircle the tumor but fail to infiltrate the tumor parenchyma, undermine their anti-tumor effects (41). The relationship between anoikis and immune cell infiltration into the TME needs further study.

To analyze the gene signature of DEGs, we identified three geneClusters with distinct clinicopathological characteristics. We observed that the geneCluster C was associated with more survival states, more Anoikisclusters C individuals, and lower stage compared to geneCluster A and B. This finding is in consistent with the longer overall survival observed in the geneCluster C. Notably, there were notable variations in the expression of anoikis regulators among the three geneClusters. We observed a low expression of DAPK2 in tumors, while its expression was relatively high in Anoikiscluster C and geneCluster C. Additionally, patients with high expression of DAPK2 exhibited a longer survival rate. It can be concluded that DAPK2 may serve as a suppressor in CRC. We also constructed and validated a predictive model based on four genes CD36, TGFβ2, WWC3, and CCL2. The efficacy of the anoikis risk score in predicting survival was demonstrated by ROC curves and the nomogram. These genes have been linked to the a tumor progression. Previous researches has indicated that TGFβ2 is connected to the prognosis of CRC and can contribute to the tumor progression of and resistance to 5-Fu (42). Moreover, CD36 plays a role in supporting metastasis by regulating lipid balance when breast cancer stromal detachment occurs (43). In this low-risk score group, we observed a high expression of immune checkpoint genes, namely CTLA4 and PD-L1. Additionally, immune-related functions analysis revealed a significant enrichment of checkpoint functions in this particular group. Recently, immunotherapy has emerged as a promising approach for the targeted treatment of CRC. Specifically, the use of immune checkpoint inhibitors (ICIs), such as CTLA4, PD-1, PD-L1, MSI-H, or dMMR, received much attention. These markers can predict the response to immunotherapy in CRC treatment (44–46). Furthermore, drug sensitivity analysis revealed that the low-risk group displayed increased susceptibility to oxaliplatin and dabrafenib. Notably, oxaliplatin serves as a frequently employed anti-CRC medication. In t CRC patients with BRAF V600E mutations, dabrafenib therapy has been associated with a potential risk of MAPK signaling reactivation. It has been documented that the combination of BRAF inhibitors and MAPK inhibitors can significantly enhance the efficacy of immunotherapy (47). Further studies are needed to treat BRAF V600E mutant CRC with BRAF inhibitors. Taken together, these results suggest that anoikis risk score can be used as an effective prognostic indicator to stratify patients with CRC and predict the response to immunotherapy regimens.

To validate DAPK2 as a prognostic indicator for CRC, we investigated the tumor-suppressive properties of DAPK2 in CRC. We found a decreased expression of DAPK2 in tumor tissues. Previous studies have highlighted the role of DAPK2 in regulating apoptosis and autophagy (22, 48, 49). Consistently, they revealed that high DAPK2 levels can facilitate apoptosis in colon cancer cells. Nevertheless, there are reports on tr effects of DAPK2 in metastasis. Jin M et al. reported that DAPK2 can inhibit the proliferation and migration of lung cancer cells (50). In this study, we discovered that the expression of DAPK2 is lower in patients with T1-3 tumors than in patients with T4 tumors, suggesting that the expression of DAPK2 is related to the local tumor progression and metastasis. Consistently, we demonstrated that DAPK2 can inhibit the CRC cell growth and metastasis and promote CRC cell apoptosis. AKT1 plays a key role in regulating cell proliferation and migration. Inhibition of AKT phosphorylation has been shown to suppress the growth and invasion of non-small cell carcinoma, breast cancer, and hepatocellular carcinoma cells (51). Our findings suggest that DAPK2 can suppress CRC cells migration by modulating the AKT1/CyclinD1 pathway. These results strongly imply that DAPK2 could offer a novel and highly specific therapeutic target for the treating CRC.

There are certain limitations within our research. First, we must acknowledge the potential presence of selection bias in the data acquired from the public dataset. There were too few clinical samples of CRC we collected to fully validate the association of DAPK2 with prognosis and clinical features. Furthermore, although we investigated the association between anoikis and TME phenotypes, further experimental studies are indispensable to fully verify the relationship. Finally, we confirmed that DAPK2 overexpression can inhibit tumor growth and metastasis, but the underlying mechanism needs further study.

## Conclusion

In conclusion, this study uncovered an innovative anoikis-related gene expression pattern, which exhibited unique clinicopathological and TME characteristics. The novel anoikis-related risk score indicated distinct prognostic signatures, clinical-pathological characteristics, immunophenotypes, and potential treatment options, thus fostering the implementation of precision medicine for CRC. The anoikis-related gene DAPK2 was shown to inhibit tumor growth and metastasis through the AKT1/CyclinD1 pathway, presenting a new target for the treating of CRC.

## Data availability statement

The original contributions presented in the study are included in the article/**Supplementary Material**. Further inquiries can be directed to the corresponding author.

## Ethics statement

The studies involving humans were approved by Ethics Committee of Shuguang Hospital Affiliated to Shanghai University of Traditional Chinese Medicine. The studies were conducted in accordance with the local legislation and institutional requirements. The participants provided their written informed consent to participate in this study. The animal study was approved by Experimental Animal Ethics Committee, Shanghai University of Traditional Chinese Medicine. The study was conducted in accordance with the local legislation and institutional requirements.

## Author contributions

CL: Conceptualization, Data curation, Formal analysis, Investigation, Methodology, Software, Visualization, Writing – original draft, Writing – review & editing. JW: Investigation, Methodology, Supervision, Visualization, Writing – review & editing. LY: Conceptualization, Supervision, Visualization, Writing – review & editing. HG: Formal analysis, Supervision, Visualization, Writing – review & editing. ZL: Conceptualization, Supervision, Writing – review & editing, Investigation.
