# Supplementary material for: Development of an anoikis-related gene signature and prognostic model for predicting the tumor microenvironment and response to immunotherapy in colorectal cancer
Source: Front Immunol. 2024 May 8;15:1378305. doi: 10.3389/fimmu.2024.1378305 (PMC11109372; doi:10.3389/fimmu.2024.1378305)
Supplement: Supplementary file 1 [file DataSheet_1.docx]

Figure S1. Metascape analysis of 18 anoikis regulators.

Figure S2. The Venn diagram of DEGs between three Anoikisclusters.


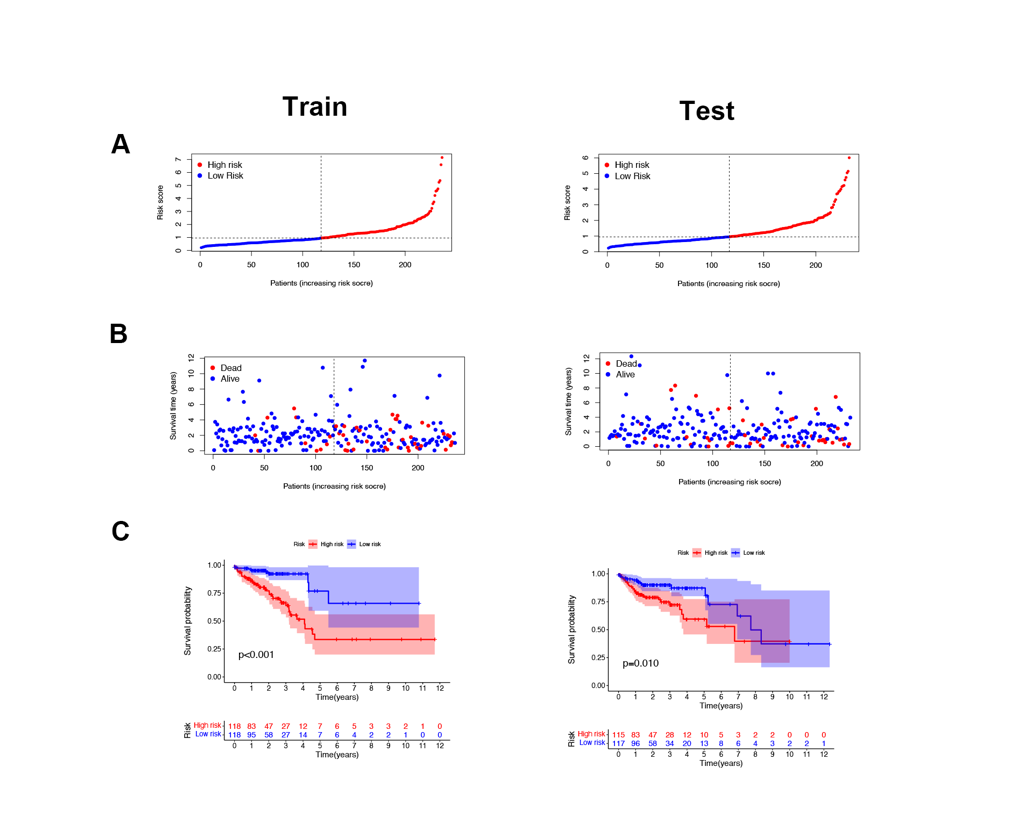


Figure S3. Prognostic value of risk scores in train set and test set.

(A) Rank dot showed different subgroups according to the median values. (B) Relationship between the survival status and risk score. (C)K-M survival analysis of patients in training and testing set.

Figure S4.Realtive expression of PDL-1 in high and low risk score groups.


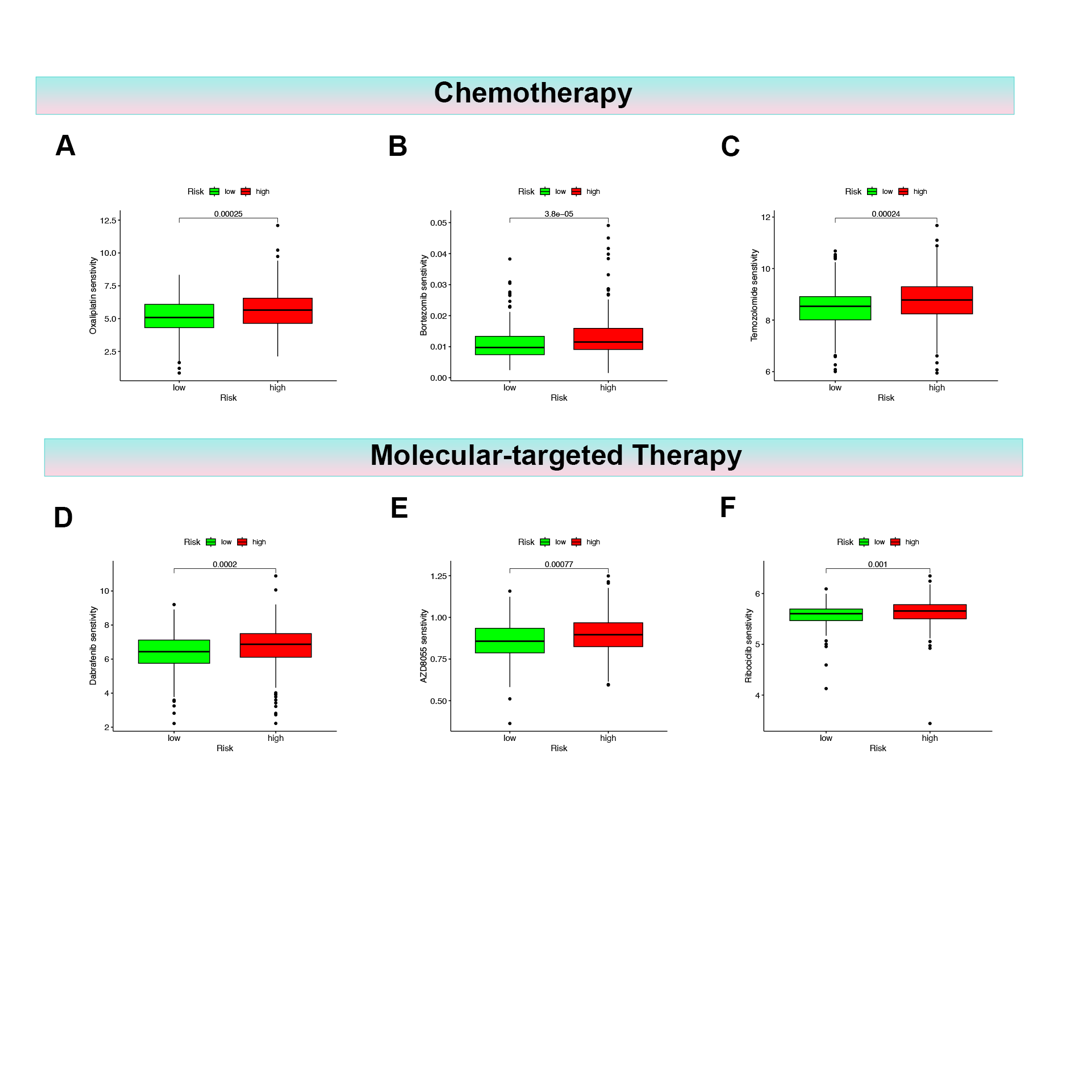


Figure S5. Drug susceptibility testing.

Relationships between the risk scores and susceptibility to chemotherapy or targeted therapies for CRC. (A) Oxaliplatin. (B) Bortezomib. (C) Temozolomide. (D) Dabrafenib. (E) AZD8055. (F) Ribociclib.
